# The causal role of genetically predicted obesity-related traits in heart failure: systematic review and meta-analysis of Mendelian randomization studies

**DOI:** 10.3389/fcvm.2026.1798689

**Published:** 2026-04-10

**Authors:** Xiuli Shi, Xuefeng Wang, Yixuan Li, Hao Luo, Yihang Li, Na Lai, Xiang Zheng, Ting Zhang, Yongqiu Zeng, Kai Guo, Ji Li

**Affiliations:** 1 School of Clinical Medicine, Xi’an Medical University, Xi’an, Shaanxi, China; 2 Second Ward of Cardiology Department, The First Affiliated Hospital of Xi'an Medical University, Xi'an, Shaanxi, China; 3 Department of Cardiology, The Affiliated Hospital, Southwest Medical University, Luzhou, Sichuan, China; 4 College of Basic Sciences, Southwest Medical University, Luzhou, Sichuan, China; 5 Department of Clinical Medicine, School of Clinical Medicine, Southwest Medical University, Luzhou, Sichuan, China

**Keywords:** heart failure, Mendelian randomization, meta-analysis, obesity, systematic review

## Abstract

**Background:**

Obesity is a major public health concern and has been implicated in the pathogenesis of heart failure (HF). However, the causal nature of this relationship remains to be comprehensively elucidated through robust genetic epidemiological approaches.

**Objective:**

This systematic review and meta-analysis aimed to synthesize evidence from Mendelian randomization (MR) studies regarding the causal effects of genetically predicted obesity and related anthropometric traits on HF and its subtypes.

**Methods:**

A comprehensive literature search was conducted across PubMed, Google Scholar, Web of Science, Embase, and the Cochrane Library for studies published up to October 2025. Study quality was assessed, and random-effects meta-analysis were performed where applicable.

**Result:**

Genetically predicted adult BMI was significantly associated with increased HF risk across European (OR_SD_: 1.79; 95% CI: 1.64–1.94), and East Asian ancestries (OR_kg/m_^2^: 2.17; 95% CI: 1.79–2.63). A significant association was also observed for HF with preserved ejection fraction (HFpEF) in European-ancestry individuals (OR_SD_: 2.68; 95% CI: 1.07–4.28). Childhood body size traits were associated with HF (OR_SD_: 1.30; 95% CI: 1.21–1.39). Fat mass, WC, WHR, and unfavorable adiposity were also identified as causal risk factors. Tissue-specific analyses indicated that both brain- and adipose-tissue-specific genetic instruments for BMI were associated with elevated HF risk.

**Conclusion:**

This study provides robust MR evidence supporting a causal role of multiple obesity-related traits, particularly adult BMI, in the development of HF across diverse populations. Further MR studies in non-European populations and investigations into specific pathways are warranted to enhance generalizability and mechanistic understanding.

**Systematic Review Registration:**

crd.york.ac.uk/PROSPERO/display_record.php?RecordID=576216 identifier, CRD42024576216.

## 1 Introduction

Heart failure (HF) remains a leading cause of global mortality, morbidity, and hospitalization, significantly impairing patients' quality of life (1). The prevalence and severity of HF continue to escalate, constituting a growing public health crisis. According to the American Heart Association's 2024 statistics, HF prevalence is projected to increase by 46% between 2012 and 2030, affecting approximately 8.5 million adults aged 20 or older in the United States (1, 2). Alarmingly, HF-attributable deaths rose by 45.8% from 2011 to 2021 (3).

Obesity, defined as abnormal or excessive fat accumulation that compromises health, was formally classified as a disease. Recent data indicate that the age-adjusted prevalence of obesity among US adults aged 20 and older surged from 22.9% to 42.2% between 1984 and 2018—an increase of 19.5% (4). Numerous observational studies and meta-analyses have consistently demonstrated a close, multifactorial pathophysiological association between obesity-related traits and HF (5). The mechanisms involved include multiple pathways: chronic volume and pressure overload lead to cardiac remodeling; inflammatory factors secreted by adipose tissue promote cardiac fibrosis and insulin resistance; additionally, comorbidities like metabolic syndrome further exacerbate cardiac damage. Epidemiological studies consistently indicate that obesity is an independent risk factor for heart failure, increasing the risk of the disease by approximately 40%–50% (6). Large cohort studies (such as the Framingham Heart Study) have confirmed that even modest to moderate weight loss—generally defined as a reduction of 5%–10% of body weight—can significantly improve cardiac function and prognosis (7). These observations raise a central question: does obesity have a causal role in HF? Although randomized controlled trials are the gold standard for establishing causality, they are often impractical for this question due to high costs, long follow-up periods, and ethical concerns, residual confounding, and potential reverse causation (8).

**Figure 1:**
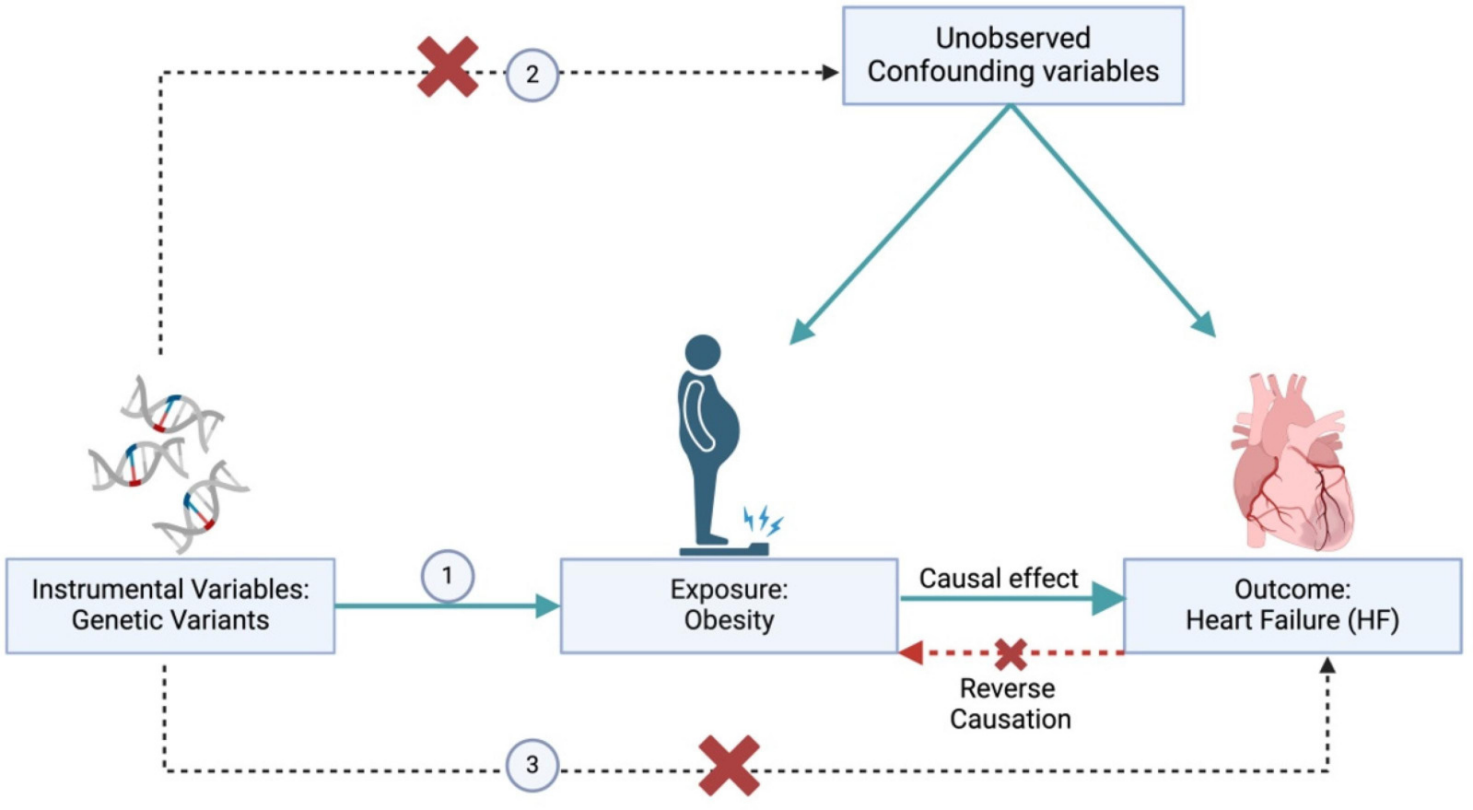
The directed acyclic graph (DAG) illustrates the assumptions of the Mendelian randomization (MR) study on the causal relationship between obesity and heart failure (HF). The assumptions are as follows: (1) genetic variants must be reliably associated with obesity; (2) genetic variants must not be associated with any known or unknown confounders; (3) genetic variants must not influence HF directly, but only through obesity.

Advances in genetics over the past decade have provided new avenues for causal inference. Large-scale genome-wide association studies (GWAS) have identified numerous genetic variants associated with obesity-related traits (9, 10) and heart failure, demonstrating that obesity directly increases the risk rather than being driven solely by confounding factors (11, 12). These discoveries have enabled Mendelian randomization (MR) analysis, an approach that uses genetic variants as instrumental variables(IVs) to assess causal relationships from observational data (8) (Figure 1). Compared with traditional observational studies, MR studies are not biased by reverse causality, since genetic variants are determined at conception and remain unaffected by subsequent disease processes or environmental exposures (13). Moreover, MR studies are less susceptible to confounding, as genetic variants are inherited independently—that is, randomly—at meiosis, mimicking the randomization process in a controlled trial (14).

Elucidating the relationship between obesity and heart failure is of significant clinical and public health importance for developing effective strategies for the prevention and treatment of HF. Therefore, this study aims to conduct a systematic review and meta-analysis of MR studies investigating the causal effect of genetically predicted obesity on the risk of heart failure.

## 2 Methods

This systematic review was conducted in accordance with the Preferred Reporting Items for Systematic Reviews and Meta-Analyses (PRISMA) guidelines (15). The review protocol was registered prospectively on the International Prospective Register of Systematic Reviews platform. (Registration number: CRD42024576216. crd.york.ac.uk/PROSPERO/display_record.php?RecordID=576216).

### 2.1 Search strategy and screening process

A systematic literature search was conducted using PubMed, Google Scholar, Web of Science, Embase, and the Cochrane Library for records published up to October 2, 2025. The search strategy used keywords and Medical Subject Headings related to heart failure (e.g., “Heart Failure”, “Cardiac Failure”, “heart decompensation”) and obesity-related traits (e.g., “adiposity”, “obesity”, “body mass index”, “body composition”, “body fat distribution”). Hand-search of the reference lists of retrieved articles, previous meta-analyses, and reviews were also performed to identify relevant publications.

Eligible studies were original MR analyses examining the causal effect of genetically predicted obesity-related traits on HF or its subtypes. To capture a broad spectrum of relevant studies based on methodological design—not merely those self-labelled as MR—we employed a search filter including key methodological terms such as “instrumental variable,” “Mendelian randomization,” and “genetic instrument”.

Two authors (XLS and JL) independently screened the titles and abstracts of all searched papers. Studies included meet the following criteria: (1) applied an MR or IVs design using genetic variants; (2) evaluated obesity-related exposures such as childhood or adult body mass index (BMI), waist circumference (WC), waist-to-hip ratio (WHR), fat mass(FM), fat-free mass(FFM), visceral adipose tissue volume(VAT), abdominal subcutaneous adipose tissue volume (ASAT), body fat percentage, favorable and unfavorable adiposity, WHR adjusted BMI, or tissue-specific IVs (e.g., adipose- or brain-specific BMI instruments); (3) assessed HF overall or its key subtypes [heart failure with preserved ejection fraction (HFpEF) or heart failure with reduce ejection fraction (HFrEF)]. Studies were excluded if they were: (1) abstracts and conference proceedings; (2) non-human studies; (3) non-English studies (to maintain consistency in data extraction and reduce language-related bias); (4) without a control group; (5) focusing exclusively on specialized treatment populations, (e.g., patients undergoing bariatric surgery or advanced heart failure therapies).

### 2.2 Data extraction and management

Two authors (XLS and XFW) independently extracted all data from the included studies using a standardized form and reached consensus on all items after discussion. When disagreements arose, a third researcher (JL) assessed the articles to resolve them. The following details were collected: first author, publication year, obesity trait, HF outcome, data sources, total sample size, number of cases and non-cases, population ancestry, number of single-nucleotide polymorphisms (SNPs) used as IVs in MR analysis, type of effect measure (e.g., odds ratio, hazard ratio), effect size with unit and 95% confidence interval, *p*-value, and whether a causal conclusion was reported. To facilitate heterogeneity assessment, the effect estimation model used in each study was also recorded. To assess potential overlaps in data sources, we examined whether the included MR studies shared GWAS summary statistics. We found that although some studies used common public datasets (e.g., UK Biobank, GIANT), the selection of IVs, analytical methods, and sample populations differed across studies. Detailed primary data sources are provided in Supplementary Tables S2, S3. In addition, authors of the selected studies were contacted to obtain further information when certain data was missing, or clarification was required.

### 2.3 Effect measures

There is significant variation in effect measures among the studies. Specifically, some studies use categorized obesity measures, such as obesity or body size, and report odds ratios (OR). Other studies use continuous obesity measures, including BMI, WC, WHR, FM, FFM, VAT, ASAT, etc. These studies report relative risk estimates such as OR, hazard ratio (HR), risk ratio (RR), or β coefficients per unit or standard deviation (SD) increase in the measure. To pool the effect sizes, we rescaled effect measures from studies using continuous BMI as exposure into a single effect measure—OR per 1 SD increase in BMI. From the publicly available characteristics data of UK Biobank participants48, we retrieved the mean and SD of BMI among four age and gender groups. Based on these data, we calculated the overall mean and SD (4.9 kg/m^2^) of BMI for all UK Biobank participants (Supplementary Table S4). For studies that used effect measures per 1 kg/m^2^ increment of BMI, we rescaled the OR to correspond to a per 1 SD increase in BMI using the formula. When applicable, the 95% confidence intervals (CI) of the OR were rescaled similarly.

### 2.4 Risk of bias and certainty of evidence

The risk of bias in included studies was evaluated using the revised Strengthening the Reporting of Mendelian Randomization Studies (STROBE-MR) guidelines (16, 17). In addition, the certainty of evidence for key associations was assessed using the GRADE framework, accounting for study limitations, imprecision, inconsistency, indirectness, publication bias, magnitude of effect, and dose-response gradient where applicable.

### 2.5 Data synthesis

Meta-analysis was conducted when two or more studies examined the same obesity measure or HF outcome. Random effect model was applied if high heterogeneity (*I*^2^ > 50%) was presented, otherwise, fixed effect model was used. Study-specific ORs were weighed via the inverse-variance method. Between-study variance (*τ*^2^) was estimated using restricted maximum likelihood, and heterogeneity was quantified with the *I*^2^ statistic. The Hartung–Knapp–Sidik–Jonkman method was used to compute 95% CIs for summary OR (18). Publication bias was assessed via funnel plots and Egger's test. Sensitivity analyses were conducted by iteratively excluding each study to evaluate the robustness of the pooled results. Statistical significance was set at *p* < 0.05.

## 3 Results

### 3.1 Literature search and study selection

**Figure 2:**
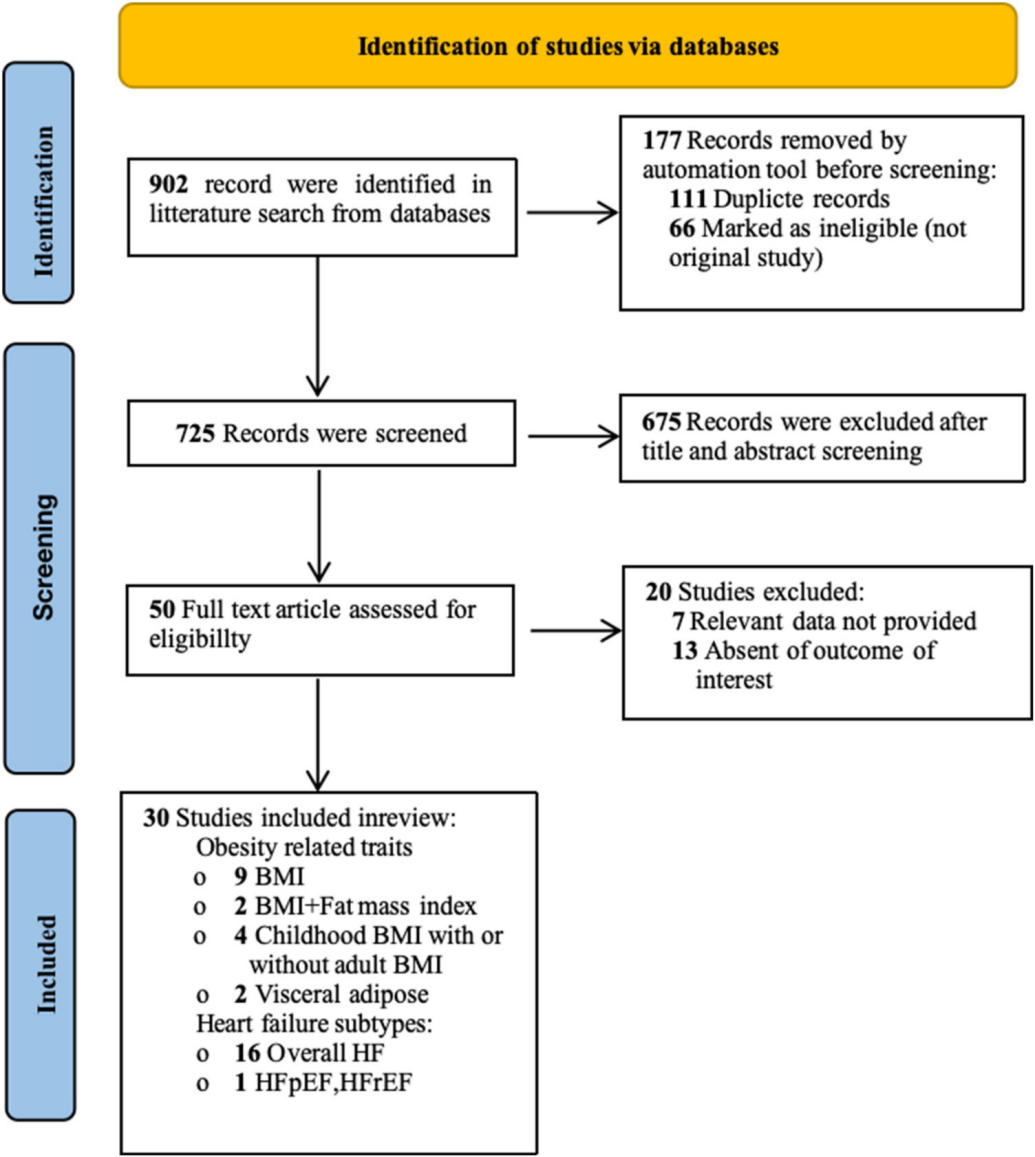
The PRISMA (preferred reporting items for systematic reviews and meta-analyses) flow diagram shows the study selection process.

The systematic search identified 902 unique records, of which 30 articles were eligible for inclusion (19–48). These studies reported MR estimates for one or more obesity-related traits in relation to HF. The study selection process is summarized in the PRISMA flow diagram, as shown in Figure 2.

### 3.2 Study characteristics and risk of bias in studies

Most studies indicated Mendelian randomization in their titles, while five studies mentioned it in the abstract. All studies reported the rationale for the study and objective and provided information on the participants and data sources used. In four studies, the numbers of cases and non-cases of HF were not found (23, 26, 39, 45). Eight studies did not mention the units used for the effect size estimates (27, 28, 31, 32, 39, 41, 42, 44).

Most studies conducted an unavoidable two-sample MR study. Some studies conducted both univariable and multivariable MR analyses. Most studies use SNPs, genetic risk scores (GRS), or polygenic scores as IVs. They obtained genetic instruments for specific obesity-related traits from one or multiple GWASs. These GWASs are based on genetic, phenotypic, clinical consortia and databases in Europe and the USA, such as GIANT, UK Biobank, FinnGen, HERMES, MVP, EGG, ARIC, FHS, MESA, and CHS etc. Most studies used ten to several hundred SNPs as IVs, while the rest of the studies used one to five selected SNPs in relevant obesity loci (e.g., FTO). Most MR studies used *P* < 5 × 10^−8^ as the threshold for statistical significance at the genomic level and R^2^ < 0.001 as the cut-off of linkage disequilibrium to extract SNPs as IVs for specific obesity-related traits. Most studies assessed the three MR assumptions, tested heterogeneity and pleiotropy, and conducted sensitivity analyses. The results of the evaluation of risk of bias can be found in the Supplementary Table S1.

Nineteen studies examined multiple obesity-related traits, while eleven studies examined one obesity-related trait. The number of MR studies based on independent study samples for each obesity-related trait was twelve for adulthood BMI, three for childhood BMI, two for WHR adjusted BMI, one for adipose and brain-tissue-instrumented BMI, four for FM, three for FFM, four for childhood BMI/body-size/obesity, four for VAT, two for ASAT, two for body fat percentage, four for WC and WHR, one for favorable and unfavorable adiposity.

Three studies examined subtypes of HF, including HFpEF and HFrEF (19, 38, 44); the rest of the studies focused on overall HF. The majority of studies included participants of European ancestry, while one study replicated the MR study in East Asian participants (26), and two studies recruited African American individuals (19, 23). The characteristics, details, and results of each included MR study are provided in the Supplementary Tables S2, S3).

### 3.3 Genetically predicted adult BMI

**Figure 3:**
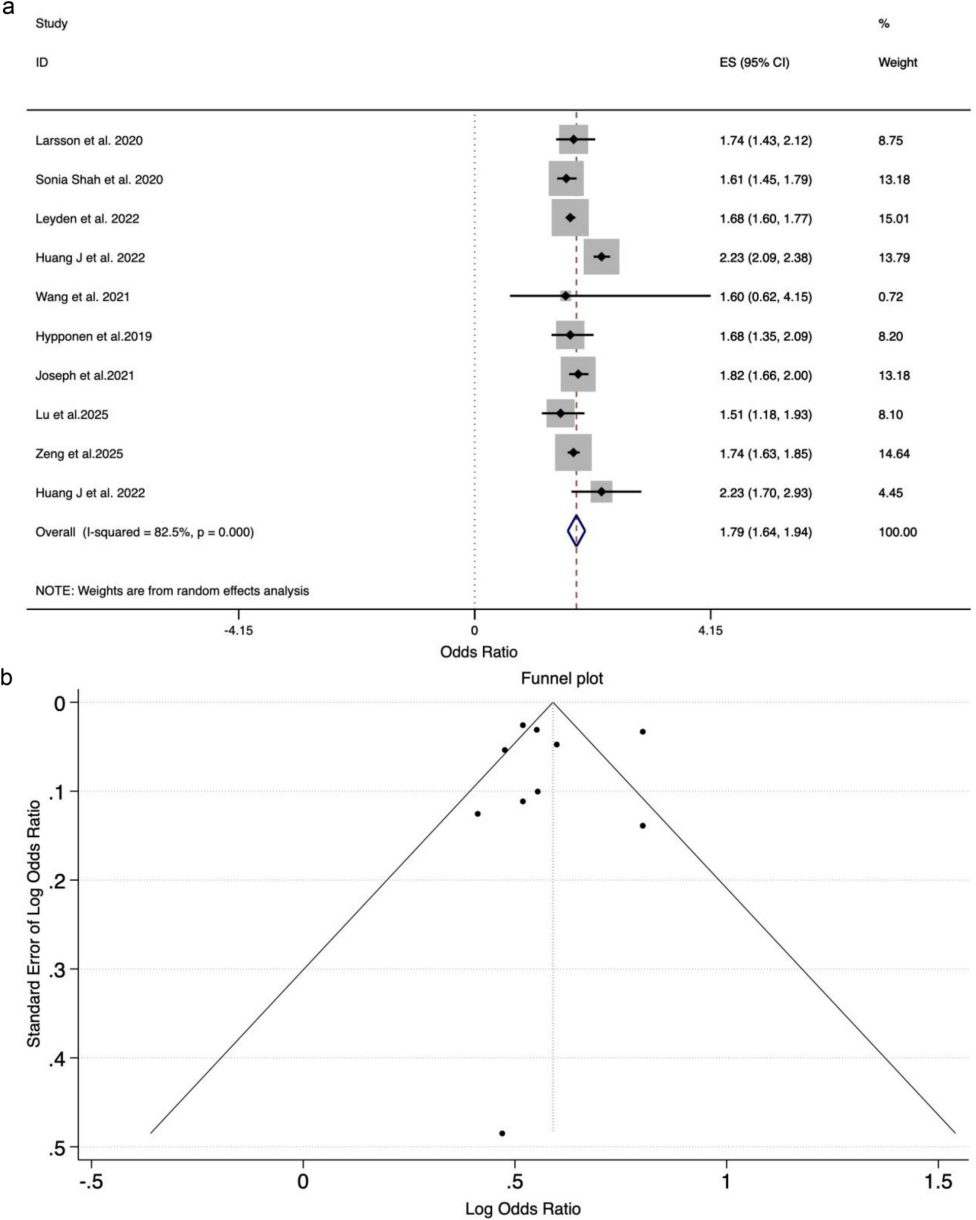
**(a)** Meta-analysis of the association between genetically predicted adult BMI and HF using a random-effects model in European and African ancestry. **(b)** Funnel plot showing no significant publication bias in the meta-analysis of genetically predicted adult BMI and HF.

Among all obesity-related measures investigated in published MR studies, genetically predicted adult BMI—a measure of general obesity—has the strongest association with the risk of HF. This effect remained significant in multivariable MR studies after controlling other risk factors. Specifically, ten studies comprising a total of 201,903 cases, and 3,533,620, non-cases genetically predicted adult BMI was associated with a higher risk of HF in individuals of European and African ancestry (combined OR_SD_ = 1.79, 95% CI: 1.64–1.94) [Figure 3(a)], and East Asian ancestry (ORkg/m^2^ = 2.17, 95% CI: 1.79–2.63). A causal effect between BMI and HFpEF was observed in European ancestry (OR_SD_ = 2.68, 95% CI: 1.07–4.28) but not in African ancestry (OR_SD_ = 1.88, 95% CI: 0.88–4.03) (Supplementary Table S2).

Significant heterogeneity was observed among the studies, with *I*^2^ = 82.5% and H^2^ = 5.71. Despite this heterogeneity, the leave-one-out sensitivity analysis showed that no single study changed the effect estimate significantly, confirming result robustness. The funnel plot [Figure 3(b)] is roughly symmetric, and this symmetry is confirmed by a non-significant result from Egger's test (*p* = 0.891), suggesting no indication of publication bias in this analysis. Four articles conducted two-sample bidirectional MR studies on the causal relationship between BMI and HF and found that BMI was an independent indicator of HF development (27, 31, 32, 41). However, the unit of the effect measure was not reported in these studies. As a result, two of these studies were excluded from meta-analysis.

### 3.4 Childhood BMI/body size/obesity

Six univariable MR studies examined genetically predicted childhood obesity measures, such as childhood BMI, childhood body size, and childhood obesity. These studies indicated a causal association between these measures and the risk of HF (Supplementary Table S2). For example, three studies comprising a total of 90,068 cases, and 1,482,877, non-cases observed a causal association between childhood BMI and HF (OR_SD_ = 1.30, 95% CI: 1.21–1.39). There is no significant heterogeneity among studies, with an *I*^2^ of 0% and an H^2^ of 0.065. Leave-one-out sensitivity analysis confirmed the robustness of our findings. The symmetric funnel plot and non-significant Egger's test (*p* = 0.295) indicated no publication bias.

Two studies reported findings on the association between childhood obesity and heart failure using OR, although with differences in the reporting of effect measure units. One study did not specify or define the units for the effect measure but reported an OR of 1.11 (95% CI: 1.05–1.17), indicating a statistically significant positive association (35). The other study defined the exposure as a 1-unit increase in log-odds and reported an OR of 1.10 (95% CI: 1.03–1.18), also showing a statistically significant positive association (30). Given the variation in units of effect measure, and the lack of clarity in one study regarding the specific increment in exposure, direct comparison or quantitative synthesis of these results is not feasible.

**Figure 4:**
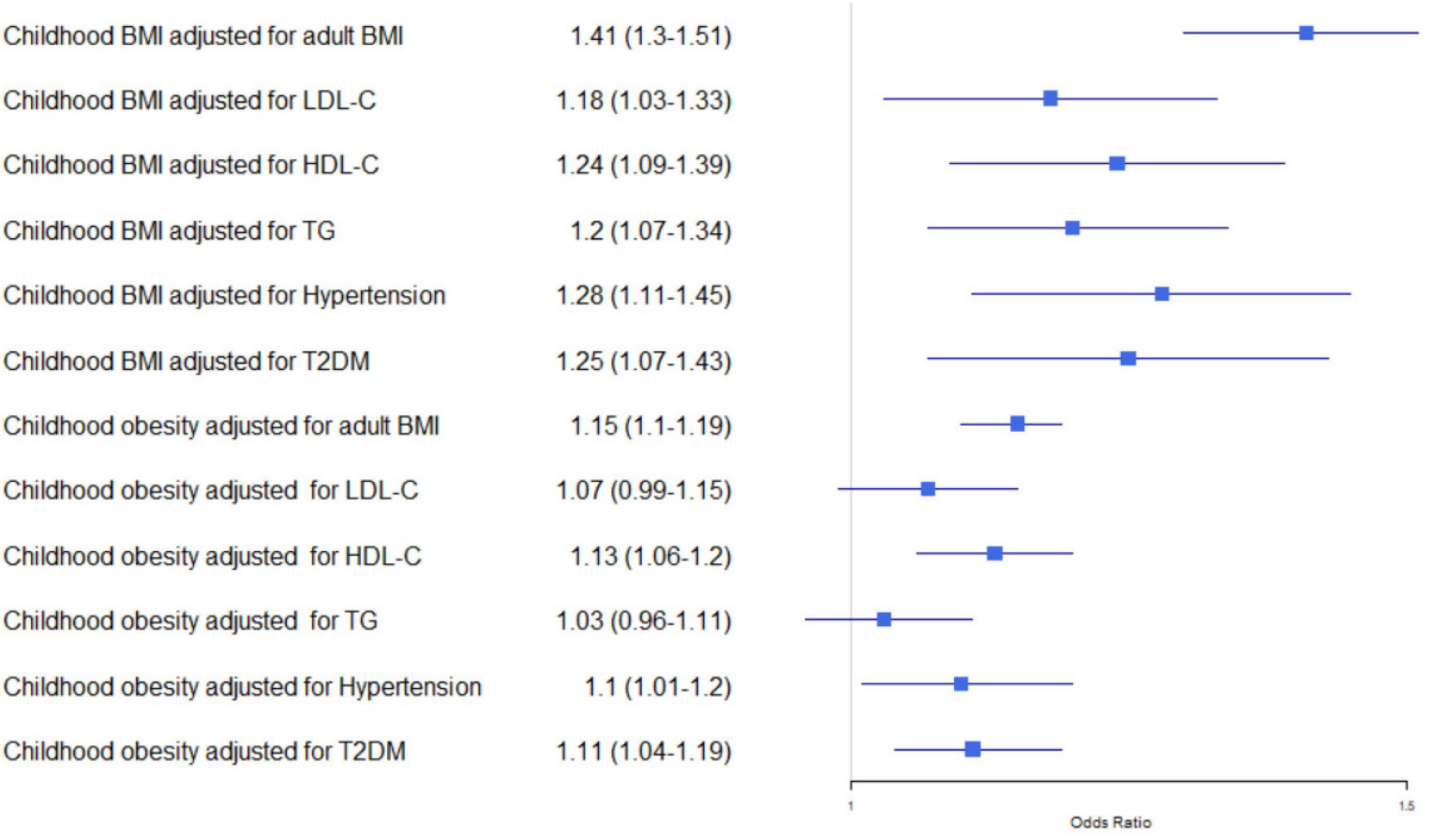
Multivariable Mendelian randomization analyses examined associations of childhood BMI and childhood obesity with HF, adjusting for cardiometabolic risk factors (30). BMI, body mass index; HDL-C, high-density lipoprotein cholesterol; LDL-C, low-density lipoprotein cholesterol; T2DM, type 2 diabetes; TG, triglycerides.

However, in Multivariable MR analyses that some studies conducted, the causal associations between childhood obesity with HF was mixed after controlling for cardiometabolic factors. Li et al. found that childhood BMI was associated with an increased risk of chronic heart failure (OR _Kg/m_^2^ = 1.33, 95% CI: 1.15–1.52, *P* < 0.001); however, this effect was attenuated to non-significance after controlling for adult BMI (26). Similarly, Martin et al. conducted Multivariable MR analyses to investigate the association between childhood body size and HF risk and found that the significant association disappeared after adjusting for adult body size (27). On the other hand, Xiong et al. found that both childhood BMI and childhood obesity were associated with HF in univariable MR analysis (Supplementary Table S2). This association remained significant in multivariable MR analysis after adjustment for specific adult cardiometabolic risk factors such as LDL-C, HDL-C, TG, hypertension, and T2DM, particularly after adjustment for adult BMI, with odds ratios of 1.41 (95% CI: 1.30–1.51) for childhood BMI and 1.15 (95% CI: 1.10–1.19) for childhood obesity, respectively (Figure 4) (30). Notably, Xiong's results showed a strong association between childhood BMI and HF after controlling for adult BMI, which contrasts with the findings of Li and Martin.

### 3.5 Fat mass and fat-free mass

Four MR studies evaluated the effect of fat mass on HF risk. All observed a significant association in both univariable and multivariable MR analyses (Supplementary Tables S2-S3) (24, 31, 42, 46). Two of these studies did not report the units used for the effect measure. Hence, only two studies comprising a total of 28,200 cases, and 557,711, non-cases were included to synthesize the findings (OR_SD_ = 1.36, 95% CI: 1.01–1.72). There was no significant heterogeneity among these studies, with an *I*^2^ of 38.5% and H^2^ of 1.63.

Three studies investigated the effect of fat-free mass on HF risk. All of them assessed the association using univariable MR analyses. One study did not report the units used for the effect estimate. Therefore, only two studies comprising a total of 28,200 cases, and 557,711, non-cases were included to synthesize the findings (OR_SD_ = 1.09, 95% CI: 0.45–1.73). One of these two studies also conducted a multivariable MR analysis. However, the effect was also not significant after adjusting for either BMI or WC (Supplementary Table S3).

### 3.6 Visceral adipose tissue (VAT) and abdominal subcutaneous adipose tissue volume (ASAT)

**Figure 5:**
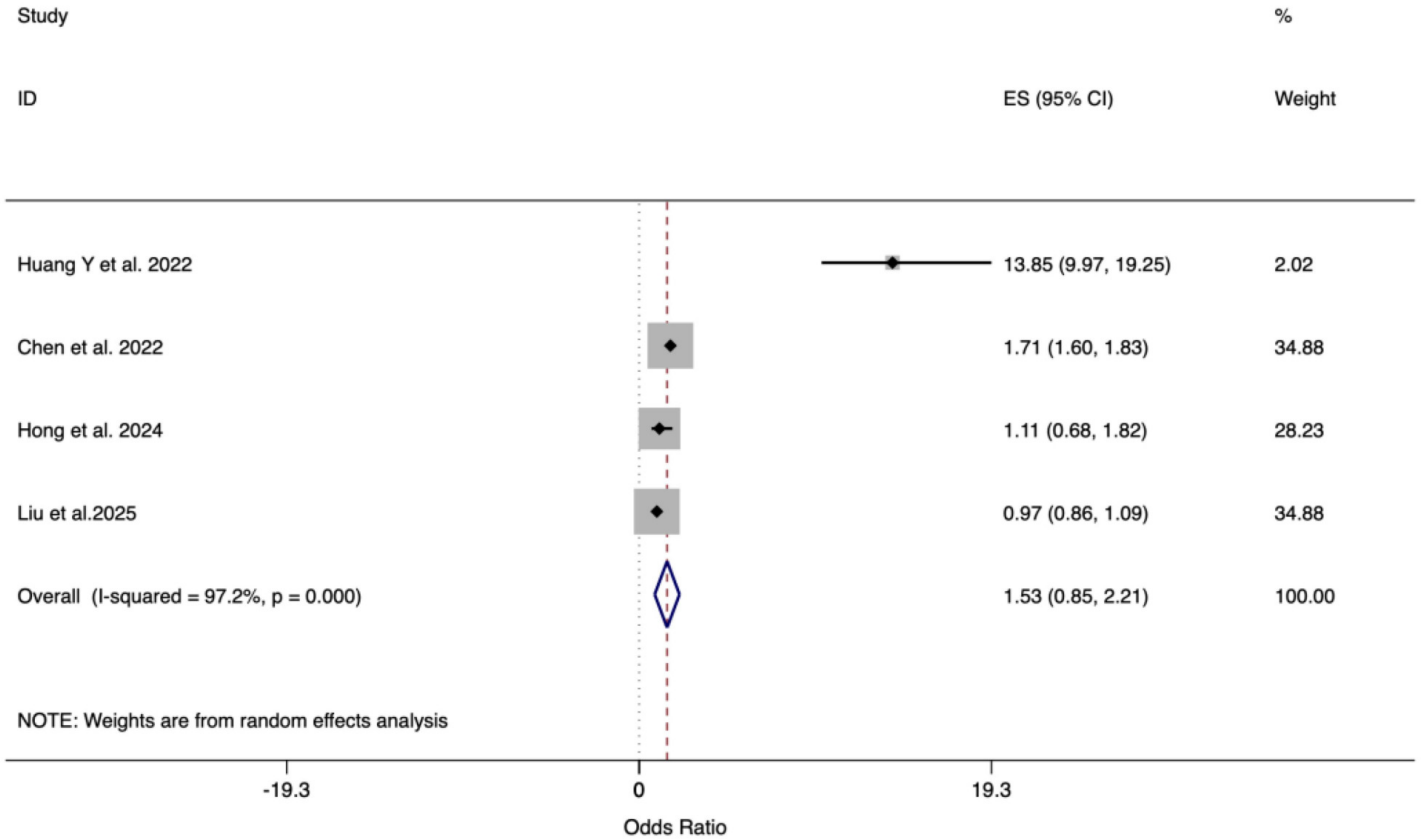
Meta-analysis on the association between VAT and HF using random-effect model.

Four studies comprising a total of 141,927 cases, and 2,790,042 non-cases investigated the causal association between genetically predicted VAT and HF risk. All of them conducted univariable MR analyses. The results of the meta-analysis showed no significant association between VAT and HF (OR = 1.53, 95% CI: 0.85–2.21) (Figure 5). There was significant heterogeneity among the studies, with *I*^2^ of 97.2% and H^2^ of 36.02, therefore a random-effects model was applied. Leave-one-out sensitivity analysis confirmed the robustness of these findings. The symmetric funnel plot and a non-significant Egger's test (*p* = 0.695) indicated no publication bias. One of the four studies also investigated the causal association using multivariable analyses, which showed no significant association (Supplementary Table S2).

Two studies investigated the effect of ASAT on the risk of HF using both univariable and multivariable analyses, and their results suggested that ASAT was not associated with an increased risk of HF.

### 3.7 Waist circumference and waist-hip-ratio

**Figure 6:**
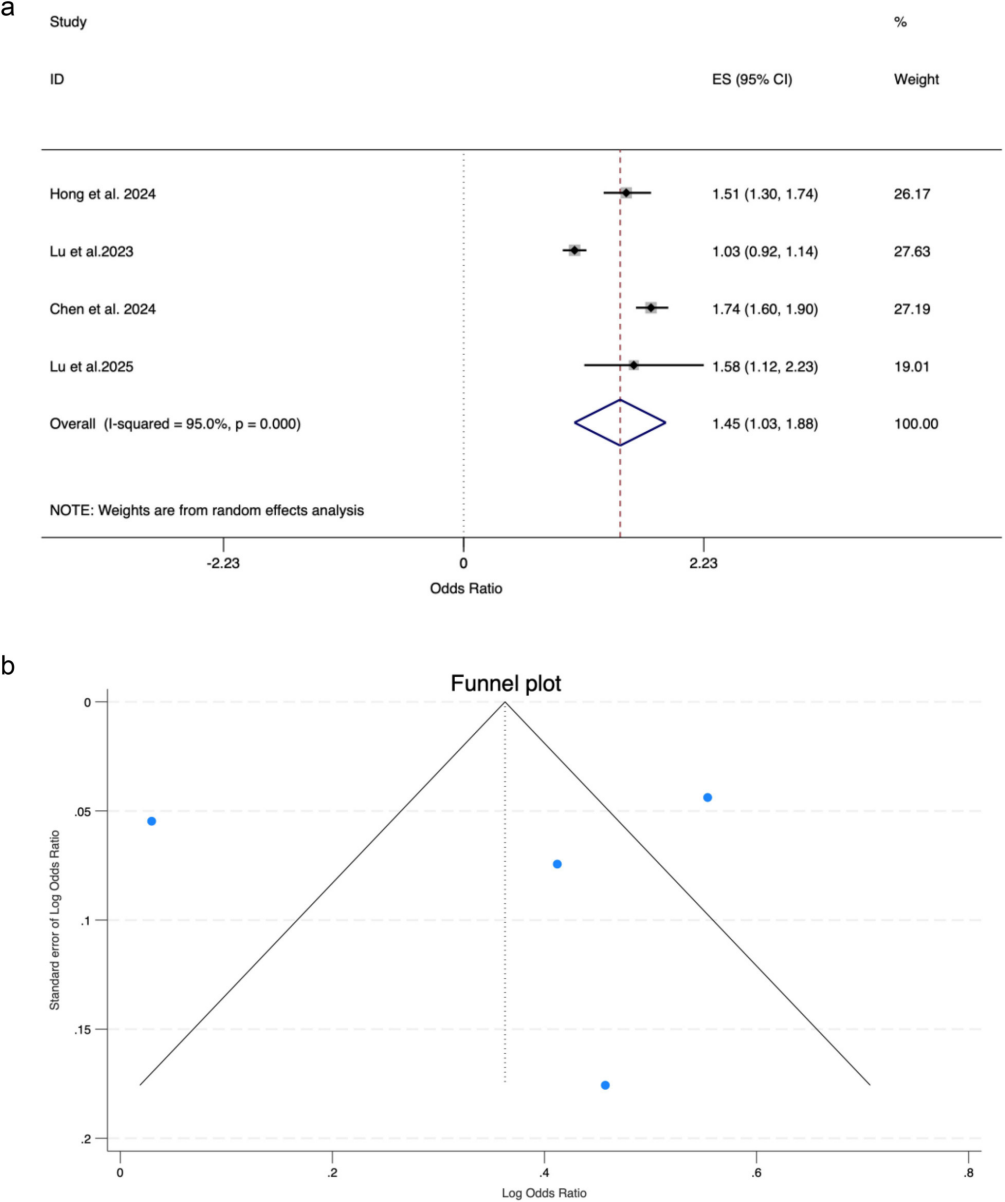
**(a)** Meta-analysis of the association between waist circumference and HF using a random-effects model. **(b)** Funnel plot showing no significant publication bias in the meta-analysis of waist circumference and HF.

Four studies comprising a total of 165,324 cases, and 2,984,853 non-cases investigated the causal association between WC and HF risk. All of them conducted univariable MR analyses. Results of the meta-analysis showed an overall significant association between WC and HF (OR_SD_ = 1.45, 95% CI: 1.03–1.88) [Figure 6(a)]. Significant heterogeneity was observed among the studies, with *I*^2^ = 95.0% and H^2^ = 20.17. Despite this heterogeneity, the leave-one-out sensitivity analysis confirmed the robustness of our findings. The symmetric funnel plot and non-significant Egger's test (*p* = 0.901) indicated no publication bias [Figure 6(b)]. Two of the four studies also investigated the causal association using multivariable analyses, which was attenuated to null (Supplementary Table S3).

Four studies comprising a total of 118,015 cases, and 2,054,839 non-cases investigated the causal association between WHR and HF risk. Results of the meta-analysis showed an overall significant association between WHR and HF (OR = 1.19, 95% CI: 1.03–1.34). Two studies investigated the causal association between WHR adjusted for BMI and HF risk; however, both results were not significant. Significant heterogeneity was observed among the studies, with I² of 81.5% and H² of 5.42. Despite this heterogeneity, the leave-one-out sensitivity analysis confirmed the robustness of our findings. The symmetric funnel plot and non-significant Egger's test (*p* = 0.208) indicated no publication bias. However, two studies also investigated the causal association using multivariable analyses, which was attenuated to null (Supplementary Table S3).

### 3.8 Favorable/unfavorable adiposity

MR studies on favorable and unfavorable adiposity are scarce, with only one MR study involving 47,309 cases, and 930,014 non-cases to investigate the effect of adiposity on HF risk (27). The results suggested that favorable adiposity is not associated with HF risk, whereas unfavorable adiposity significantly increases the risk (OR = 2.29, 95% CI: 1.85–2.83). The study identified 36 genetic variants associated with “favorable adiposity”. These variants are associated with a favorable metabolic profile characterized by higher subcutaneous fat and lower ectopic liver fat, resembling a polygenic phenotype opposite of lipodystrophy. They also identified 38 “unfavorable adiposity” variants, which are associated with higher subcutaneous and visceral fat, as well as increased ectopic liver and pancreatic fat, resembling a phenotype like monogenic obesity.

### 3.9 Brain-tissue-instrumented BMI/adipose-tissue-instrumented BMI

Leyden et al. identified variants associated with brain and adipose tissue. They then conducted a two-sample MR study involving 10,155 cases, and 324,243 non-cases to investigate the effect of BMI on HF risk (25). The results showed that both brain-tissue-instrumented BMI and adipose-tissue-instrumented BMI were strongly associated with an increased risk of HF (Supplementary Table S2).

## 4 Discussion

### 4.1 Primary findings

Overall, the meta-analysis of MR studies on genetically predicted obesity-related traits and HF provides evidence for consistent causal associations. Specifically, genetically predicted adult BMI is associated with an increased risk of HF in European, African, and East Asian populations. This finding aligns with the conclusion from large-scale genetic studies that position BMI as a central node in a broad network of diseases (19), reinforcing the public health strategy of placing obesity management at the core of primary prevention for heart failure. Future MR replication studies in diverse population groups are needed to confirm the external validity of these findings.

However, an in-depth analysis of the relationship between different obesity phenotypes and HF subtypes reveals the complexity of existing evidence and future research directions. First, conflicting results have been reported regarding adult BMI and its relation to HFpEF. This discordance may stem from genuine biological heterogeneity or may be related to the efficacy or pleiotropic characteristics of IVs in different ancestral populations. Due to the lack of additional studies evaluating such associations, future replication MR studies are needed to provide robust evidence of the relationship between genetically predicted BMI and HFpEF risk, a condition more challenging to diagnose, treatment, and manage than HFrEF (49). Furthermore, there is a scarcity of studies assessing this relationship among African Americans, who bear a disproportionate burden of both obesity and heart failure. In the USA, African Americans have the highest prevalence and hospitalization rates for HF, and the highest rates of obesity and severe obesity (BMI ≥ 40) compared to other racial groups (50, 51). Confirming the causal role of obesity in heart failure among African Americans is critical for developing preventive strategies, personalized medicine, and other targeted approaches. Variation in effect measures across individual MR studies indicates inconsistency in current research, complicating the interpretation, utilization, and comparison of findings. We therefore call for future research to use uniform effect measures, such as odds ratios, whenever applicable. Furthermore, future studies targeting populations with a high burden of HF and obesity, such as African Americans (19), must focus on constructing more precise, ancestry-specific genetic tools, supplemented by more rigorous pleiotropy testing.

Childhood-related obesity traits, such as childhood BMI, childhood body size, and childhood obesity, are causally associated with an increased risk of developing heart failure. However, it remains inconclusive whether this association results from larger body size persisting into adulthood, since conflicting results were found after controlling for adulthood BMI or other obesity-related factors. However, the risk for HF is multifactorial, and factors beyond adult BMI—which themselves may be associated with early-life BMI—might confound or mediate the relationship. Solely controlling for adult BMI may be insufficient to fully reveal the independent effect of childhood BMI. The significant association observed by Xiong et al. after controlling for adult BMI may precisely capture these effects not fully mediated by adult BMI (30). Therefore, future investigations into the causal impact of early-life obesity on HF would benefit from considering these multidimensional factors alongside adult BMI to more precisely elucidate its independent mechanisms and to ascertain whether childhood-related obesity traits indirectly increase the risk of HF through a pathway involving adulthood BMI.

MR studies showed that general adiposity is more strongly associated with HF risk than central adiposity, such as WC and WHR. However, these findings conflict with evidence from some observational epidemiological studies. For example, in a retrospective study of UK Biobank participants, HF risk was found to be more strongly associated with central adiposity. Specifically, WC adjusted for BMI had a hazard ratio (HR) of 1.38 (95% CI 1.32–1.45), while BMI adjusted for WC had an HR of 1.22 (95% CI 1.16–1.27) (52). This discrepancy may stem from MR estimating the lifelong exposure effects determined by genetics, whereas observational studies are susceptible to measurement errors, reverse causality, and confounding factors that cannot be fully controlled. Future studies need to utilize more precise genetic tools for body fat distribution and aim to resolve these discrepancies to clarify how various forms and distributions of adiposity relate to heart failure risk (19). To date, only one MR study has examined the relationships between favorable or unfavorable adiposity, brain/adipose tissue instrumented BMI, and heart failure; therefore, future MR studies are needed to replicate and confirm these findings.

Our MR analysis did not find a statistically significant direct causal effect of visceral adiposity on HF risk (OR = 1.53, 95% CI: 0.85–2.21). However, this null result does not rule out the existence of important indirect pathways. The notably wide confidence interval likely reflects substantial heterogeneity and imprecision caused by methodological discrepancies across the included studies. These discrepancies include differences in units of effect measurement, a wide range in the number of genetic instruments used, and incomplete reporting of case and non-case data in one study. This aligns with the hypothesis that the pathophysiological impact of visceral adiposity may be largely mediated through intermediate phenotypes like hypertension, driven by sympathetic over activation and chronic inflammation, rather than acting as a direct independent driver of HF in a genetically predicted model (53). Prior work has shown that obesity-induced sympathetic activation promotes sodium retention and vascular resistance, fostering a hypertensive state that is a well-established direct cause of HF (53). Similarly, adipose tissue-derived cytokines (e.g., IL-1β, IL-6) contribute to a systemic inflammatory milieu that can directly impair myocardial function and promote fibrosis (54). Our results may reflect the specific methodological approach of MR, which isolates the direct lifelong effect of exposure, potentially bypassing later-onset mediators like neurohormonal and immunologic changes that play key roles in the progression from obesity to HF. Future studies should employ multivariable MR frameworks to disentangle the independent effects of visceral adiposity from its downstream consequences and investigate genetically proxied mediators like catecholamine levels or specific inflammatory markers to formally test these hypothesized pathways. Larger GWASs for precise body fat distribution phenotypes will be essential to enhance the power of causal inference.

### 4.2 Limitations

This systematic review and meta-analysis has several limitations that need to be considered. First, the statistical power of meta-analyses is limited by small sample sizes. For some obesity-related traits, only a few MR studies are available regarding their relationship with HF. Second, most MR studies used data from GWAS datasets of European ancestry; therefore, confirming the external validity of these findings in populations of different ancestries is necessary. Third, the inclusion of studies in the meta-analysis was occasionally limited by inconsistent reporting of effect size units, which poses challenges for data synthesis, despite our efforts to compensate through standardized conversion. Furthermore, some MR studies show evidence of pleiotropy, meaning a genetic variant affects multiple traits, and confounding may be present, potentially compromising the validity of causal inference in MR studies. These limitations precisely point to directions for improvement in future research: carrying out larger-scale, multi-ancestry GWASs to obtain more powerful genetic instruments; strictly adhering to standardized reporting guidelines, such as the STROBE-MR, by reporting sample size, participant ancestry, GWAS source, instrumental variable criteria, effect size unit, sensitivity analysis results, and covariates in multivariate analyses; actively conducting replication MR studies on different HF subtypes, especially HFpEF; exploring the impact of the genetic trajectory of BMI throughout the life course, as well as the genetic interactions between obesity and other cardiometabolic risk factors (such as diabetes mellitus), on the risk of HF.

## 5 Conclusions

The totality of the evidence from published MR studies and meta-analyses supports a causal role of higher adults BMI in as a risk factor for heart failure. Consequently, this finding underscores the importance of effectively controlling overweight and obesity as part of heart failure prevention strategies.

## Data Availability

The original contributions presented in the study are included in the article/Supplementary Material, further inquiries can be directed to the corresponding author.
