# Supplementary material for: The causal role of genetically predicted obesity-related traits in heart failure: systematic review and meta-analysis of Mendelian randomization studies
Source: Front Cardiovasc Med. 2026 Apr 10;13:1798689. doi: 10.3389/fcvm.2026.1798689 (PMC13105879; doi:10.3389/fcvm.2026.1798689)
Supplement: Supplementary file 1 [file Datasheet1.pdf]

Table 1: PRISMA checklist

Section and Topic	Item #	Checklist item	Location where item is reported
TITLE			
Title	1	Identify the report as a systematic review.	P1
ABSTRACT			
Abstract	2	See the PRISMA 2020 for Abstracts checklist.	P2
INTRODUCTION			
Rationale	3	Describe the rationale for the review in the context of existing knowledge.	P4
Objectives	4	Provide an explicit statement of the objective(s) or question(s) the review addresses.	P5
METHODS			
Eligibility criteria	5	Specify the inclusion and exclusion criteria for the review and how studies were grouped for the syntheses.	P6
Information sources	6	Specify all databases, registers, websites, organisations, reference lists and other sources searched or consulted to identify studies. Specify the date when each source was last searched or consulted.	P6
Search strategy	7	Present the full search strategies for all databases, registers and websites, including any filters and limits used.	P6
Selection process	8	Specify the methods used to decide whether a study met the inclusion criteria of the review, including how many reviewers screened each record and each report retrieved, whether they worked independently, and if applicable, details of automation tools used in the process.	P6
Data collection process	9	Specify the methods used to collect data from reports, including how many reviewers collected data from each report, whether they worked independently, any processes for obtaining or confirming data from study investigators, and if applicable, details of automation tools used in the process.	P7
Data items	10a	List and define all outcomes for which data were sought. Specify whether all results that were compatible with each outcome domain in each study were sought (e.g. for all measures, time points, analyses), and if not, the methods used to decide which results to collect.	P6
	10b	List and define all other variables for which data were sought (e.g. participant and intervention characteristics, funding sources). Describe any assumptions made about any missing or unclear information.	P7
Study risk of bias assessment	11	Specify the methods used to assess risk of bias in the included studies, including details of the tool(s) used, how many reviewers assessed each study and whether they worked independently, and if applicable, details of automation tools used in the process.	P7
Effect measures	12	Specify for each outcome the effect measure(s) (e.g. risk ratio, mean difference) used in the synthesis or presentation of results.	P7
Synthesis methods	13a	Describe the processes used to decide which studies were eligible for each synthesis (e.g. tabulating the study intervention characteristics and comparing against the planned groups for each synthesis (item #5)).	P8
	13b	Describe any methods required to prepare the data for presentation or synthesis, such as handling of missing summary statistics, or data conversions.	P8
	13c	Describe any methods used to tabulate or visually display results of individual studies and syntheses.	P8

Synthesis methods	13d	Describe any methods used to synthesize results and provide a rationale for the choice(s). If meta-analysis was performed, describe the model(s), method(s) to identify the presence and extent of statistical heterogeneity, and software package(s) used.	P8
	13e	Describe any methods used to explore possible causes of heterogeneity among study results (e.g. subgroup analysis, meta-regression).	P8
	13f	Describe any sensitivity analyses conducted to assess robustness of the synthesized results.	P8
Reporting bias assessment	14	Describe any methods used to assess risk of bias due to missing results in a synthesis (arising from reporting biases).	P8
Certainty assessment	15	Describe any methods used to assess certainty (or confidence) in the body of evidence for an outcome.	P8
RESULTS			
Study selection	16a	Describe the results of the search and selection process, from the number of records identified in the search to the number of studies included in the review, ideally using a flow diagram.	P8-10
	16b	Cite studies that might appear to meet the inclusion criteria, but which were excluded, and explain why they were excluded.	P10
Study characteristics	17	Cite each included study and present its characteristics.	P8-11
Risk of bias in studies	18	Present assessments of risk of bias for each included study.	P8
Results of individual studies	19	For all outcomes, present, for each study: (a) summary statistics for each group (where appropriate) and (b) an effect estimate and its precision (e.g. confidence/credible interval), ideally using structured tables or plots.	Supplementary data: Table3-4
Results of syntheses	20a	For each synthesis, briefly summarise the characteristics and risk of bias among contributing studies.	P11-16
	20b	Present results of all statistical syntheses conducted. If meta-analysis was done, present for each the summary estimate and its precision (e.g. confidence/credible interval) and measures of statistical heterogeneity. If comparing groups, describe the direction of the effect.	P11-16
	20c	Present results of all investigations of possible causes of heterogeneity among study results.	P11-16
	20d	Present results of all sensitivity analyses conducted to assess the robustness of the synthesized results.	P11-16
Reporting biases	21	Present assessments of risk of bias due to missing results (arising from reporting biases) for each synthesis assessed.	P10-11
Certainty of evidence	22	Present assessments of certainty (or confidence) in the body of evidence for each outcome assessed.	P10-11
DISCUSSION			
Discussion	23a	Provide a general interpretation of the results in the context of other evidence.	P17-19
	23b	Discuss any limitations of the evidence included in the review.	P19
	23c	Discuss any limitations of the review processes used.	P19
	23d	Discuss implications of the results for practice, policy, and future research.	P17-19
OTHER INFORMATION			
Registration and protocol	24a	Provide registration information for the review, including register name and registration number, or state that the review was not registered.	P6
	24b	Indicate where the review protocol can be accessed, or state that a protocol was not prepared.	P6
	24c	Describe and explain any amendments to information provided at registration or in the protocol.	P6

Support	25	Describe sources of financial or non-financial support for the review, and the role of the funders or sponsors in the review.	P20
Competing interests	26	Declare any competing interests of review authors.	P20
Availability of data, code and other materials	27	Report which of the following are publicly available and where they can be found: template data collection forms; data extracted from included studies; data used for all analyses; analytic code; any other materials used in the review.	P20

Table 2: Risk of bias assessment based on STROBE-MR guidelines

Study	1. Title & abstract	2. Background & objective	3. Study design & data sources	4. Study sample	5. Selection of genetic variants	6. Primary analysis	7. Sensitivity analyses	8. Software	9. Data presentation	10. Limitations & interpretation
Benn et al. 2023	1	1	1	1	1	1	1	1	1	1
Fall et al. 2013	1	1	1	1	1	1	1	1	1	1
Hagg et al. 2015	1	1	1	1	1	1	1	1	1	1
Larsson et al. 2020	1	1	1	1	1	1	1	1	1	1
Sonia Shah et al. 2020	1	1	1	1	1	1	1	1	1	-
He et al. 2018	1	1	1	-	1	1	1	1	-	1
Li et al. 2024	1	1	1	-	1	1	1	1	1	1
Kong and Li 2024	1	1	1	1	1	1	1	1	1	1
Hong et al. 2024	1	1	1	1	1	1	1	1	1	1
Xiong et al. 2024	1	1	1	1	1	1	1	1	1	1
Chen et al. 2022	1	1	1	1	1	1	1	1	1	1
Martin et al. 2022	1	1	1	1	1	1	1	1	1	1
Power et al. 2021	1	1	1	1	1	1	1	1	-	1
Leyden et al. 2022	1	1	1	1	1	1	1	1	1	1
Huang J et al. 2022	1	1	1	1	1	1	1	1	1	1
Wang et al. 2021	1	1	1	1	1	1	1	1	1	1
Huang Y et al. 2022	1	1	1	1	1	1	1	1	1	1
Hypponen et al.2019	1	1	1	1	1	1	1	1	1	1
Lumbers et al. 2020	1	1	1	1	1	1	1	1	1	1
Joseph et al.2021	1	1	1	1	1	1	1	1	1	1
Lind L et al.2021	1	1	1	-	1	1	1	1	1	1
Zhuang et al.2021	1	1	1	1	1	1	1	1	1	1
Lu et al.2023	1	1	1	1	1	1	1	1	1	1
Chen et al. 2024	1	1	1	1	1	1	1	1	1	1
Zhou et al.2024	1	1	1	1	-	1	1	1	1	1
Lin et al.2025	1	1	1	1	1	1	1	1	1	1
Liu et al.2025	1	1	1	-	1	1	1	1	1	1
Lu et al.2025	1	1	1	1	1	1	1	1	1	1
Wang et al. 2025	1	1	1	1	1	1	1	1	1	1
Zeng et al.2025	1	1	1	1	1	1	1	1	1	-

**Study quality was assessed adopting Larsson and Burgess' modified version of Strengthening the Reporting of Observational Studies in Epidemiology using Mendelian Randomization (STROBE-MR) guidelines. One point was giv*

1. *The title and/or abstract indicate Mendelian randomization design.*
2. *The background and rationale for the study and the objective are clearly reported.*
3. *The study design and data source(s) used are clearly reported.*
4. *The study sample, including the number of cases and non-cases or total number of participants included in the analysis, is reported.*
5. *The selection of genetic variants as well as the number of genetic variants used in the Mendelian randomization analysis are reported.*
6. *The statistical methods used for the primary analysis and the exposure unit are reported.]*
7. *Sensitivity analyses based on robust Mendelian randomization methods (e.g., the weighted median and/or MR-Egger regression) were conducted and reported.*
8. *The software used for the Mendelian randomization analysis is reported.*
9. *Relative risk (odds ratio) estimates are clearly presented in tables or figures.*
10. *The limitations of the study are discussed and the overall interpretation of results considering the objective and limitations is sound.*

Table 3: Study characteristics of all univariable MR studies included of systematic review

Obesity measurement	Disease Outcome	Reference	Data Sources	Cases	Non-cases	Total, N	SNPs	Effect Measur	Unit	EffectSize	LCI	UCI	P	Population	Causal Conclusi
BMI, adult	HF	Benn et al. 2023	CGPS, CCHS, GIANT, HERMES, UK Biobank	53850	1041673	1095523	5	RR	Kg/m^2	1.39	1.27	1.52	<0.001	European	Yes
BMI, adult	HF	Fall et al. 2013	ENGAGE	6068	69702	75770	1	HR	Kg/m^2	1.17	1.04	1.32	0.007	European	Yes
BMI, adult	HF	Hagg et al. 2015	ENGAGE	5649	55360	61009	32	HR	SD	1.93	1.13	3.3	0.017	European	Yes
BMI, adult	HF	arsson et al. 202	UK Biobank	4803	362900	367703	96	OR	Kg/m^2	1.12	1.07	1.16	$3.9x10^{-7}$	European	Yes
FM	HF	arsson et al. 202	UK Biobank	4803	362900	367703	82	OR	Kg/m^2	1.22	1.06	1.41	$6.3x10^{-3}$	European	Yes
FFM	HF	arsson et al. 202	UK Biobank	4803	362900	367703	82	OR	Kg/m^2	0.92	0.76	1.11	0.39	European	No
BMI, adult	HF	nia Shah et al. 2(	HERMES, UK Biobank	47309	930014	977323	89	OR	SD	1.61	1.45	1.79		European	Yes
BMI, adult	HF	He et al. 2018	ARIC, FHS, MESA, CHS, WHI			30505	32	Beta	Kg/m^2	#####			######	EA, AA	Yes
BMI, childhood	Chronic HF	Li et al. 2024	ebi-a-GCST90002409, ebi-a-GCST90018806			525780	16	OR	Kg/m^2	1.33	1.15	1.52	<0.001	European	Yes
BMI, adult	Chronic HF	Li et al. 2024	ieu-b-40, ebi-a-GCST90018806			1167435	486	OR	Kg/m^2	1.59	1.48	1.71	<0.001	European	Yes
BMI, adult	Chronic HF	Li et al. 2024	NA			342561	27	OR	Kg/m^2	2.17	1.79	2.63	<0.001	East Asian	Yes
Childhood obesity**	HF	Kong and Li 2024	EGG, HFGWAS	52839	938332	991171	13	OR		1.11	1.05	1.17	######	European	Yes
BMI, adult	HF	Hong et al. 2024	GIANT, HERMES	47309	930014	1658598	448	OR		1.74	1.63	1.85	<0.001	European	Yes
WC	HF	Hong et al. 2024	GIANT	47309	930014	1209424	38	OR		1.51	1.3	1.74	<0.001	European	Yes
WHR	HF	Hong et al. 2024	GIANT	47309	930014	1189567	28	OR		1.34	1.11	1.62	0.002	European	Yes
FM	HF	Hong et al. 2024	UK Biobank	47309	930014	1431460	360	OR		1.62	1.52	1.73	<0.001	European	Yes
FFM	HF	Hong et al. 2024	UK Biobank	47309	930014	1432173	486	OR		1.42	1.31	1.53	<0.001	European	Yes
VAT	HF	Hong et al. 2024	UK Biobank	47309	930014	1010183	5	OR		1.11	0.68	1.83	0.677	European	No
ASAT	HF	Hong et al. 2024	UK Biobank	47309	930014	1010183	2	OR		1.56	0.98	2.49	0.062	European	No
BMI, childhood	HF	Xiong et al. 2024	Vogelezang et al, 2020, FinnGen	13087	195091	247798	16	OR	SD	1.28	1.14	1.42	<0.001	European	Yes
Childhood obesity	HF	Xiong et al. 2024	EGG, FinnGen	13087	195091	222026	5	OR	1 unit increase in log-odds	1.1	1.03	1.18	<0.05	European	Yes
VAT	HF	Chen et al. 2022	HERMES	47309	930014	977323	220	OR		1.71	1.6	1.83	$.72X10^{-}$	European	Yes
BMI, adult	HF	Martin et al. 2022	GIANT	47309	930014	1269238	73	OR		1.86	1.2	2.16	######	European	Yes
BF%	HF	Martin et al. 2022	UK Biobank	47309	930014	1372292	696	OR		1.76	1.65	1.88	######	European	Yes
Favorable adiposity	HF	Martin et al. 2022	UK Biobank	47309	930014	1372292	36	OR		0.85	0.65	1.11	0.238	European	No
Unfavorable adiposity	HF	Martin et al. 2022	HERMES, UK Biobank	47309	930014	1372292	38	OR		2.29	1.85	2.83	######	European	Yes
Childhood body size†	HF	Power et al. 2021	FinnGen Study	9576	159286	168862	302	OR		1.72	1.45	2.05	$5.5x10^{-1}$	European	Yes
Adult body size†	HF	Power et al. 2021	FinnGen Study	9576	159286	168862	562	OR		2.37	2.06	2.74	<0.001	European	Yes
BMI, adult	HF	Leyden et al. 2022	GIANT, UK Biobank	10155	324243	334398	800	OR	SD	1.68	1.6	1.77	######	European	Yes
ipose-tissue-instrumented B	HF	Leyden et al. 2022	GIANT, UK Biobank	10155	324243	334398	86	OR	SD	1.08	1.06	1.11	######	European	Yes
rain-tissue-instrumented-BM	HF	Leyden et al. 2022	GIANT, UK Biobank	10155	324243	334398	140	OR	SD	1.13	1.11	1.14	######	European	Yes
BMI††	Congestive HF; nonhypertensi	uang J et al. 202	MVP, UK Biobank, GIANT	14544	144399	158943		OR	SD	2.23	2.09	2.38	######	EA***	Yes
BMI††	Congestic HF, NOS	uang J et al. 202	MVP, UK Biobank, GIANT	13414	144399	157813		OR	SD	2.26	2.11	2.41	$.62x10^{-1}$	EA***	Yes
BMI††	HFpEF	uang J et al. 202	MVP, UK Biobank, GIANT	1397	144399	145796		OR	SD	3.53	2.9	4.28	$.69x10^{-}$	EA***	Yes
BMI††	HFrEF	uang J et al. 202	MVP, UK Biobank, GIANT	1803	144399	146202		OR	SD	2.39	2.02	2.84	$.10x10^{-}$	EA***	Yes
BMI††	Congestic HF, Nonhypertensi	uang J et al. 202	MVP, AAAGC	3994	42161	46155		OR	SD	2.23	1.7	2.93	######	AA***	Yes
BMI††	HFpEF	uang J et al. 202	MVP, AAAGC	468	42161	42629		OR	SD	1.88	0.88	4.03	######	AA***	No
BMI††	Congestic HF,	uang J et al. 202	MVP, AAAGC	3714	42161	45875		OR	SD	2.46	1.85	3.26	######	AA***	Yes
BMI††	HF	Wang et al. 2021	CHARGE-HF	2526	18400	20926	77	OR	Kg/m^2	1.1	0.99	1.46	0.065	European	No
BF%	HF	Wang et al. 2021	CHARGE-HF	2526	18400	20926	12	OR	Kg/m^2	1.46	0.75	2.82	0.265	Trans-ethnic	No
WHR adj BMI	HF	Wang et al. 2021	CHARGE-HF	2526	18400	20926	39	OR	Kg/m^2	0.9	0.62	1.31	0.588	European	No
VAT	HF	uang Y et al. 202	HERMES	47309	930014	977323	219	OR	Kg/m^2	1.71	1.6	1.83	######	European	Yes
BMI, adult	Congestive HF; nonhypertensive	Hypponen et al.2019	UK Biobank	4522	327735	332257	76	OR	SD	1.68	1.35	2.09	######	European	Yes
BMI, adult	Congestive HF NOS	Hypponen et al.2019	UK Biobank	2475	327735	330210	76	OR	SD	1.74	1.33	2.28	######	European	Yes
BMI, adult	HF NOS	Hypponen et	UK Biobank	2824	327735	330559	76	OR	SD	1.7	1.3	2.23	######	European	Yes
BMI, adult	HF	Joseph et	MVP, UK Biobank	43344	258943	302287	71	OR	SD	1.82	1.66	2	######	EA***	Yes
BMI, adult	HFpEF	Joseph et	MVP, UK Biobank	19589	258943	278532	71	OR	SD	1.89	1.7	2.09	######	EA***	Yes
BMI, adult	HFrEF	Joseph et	MVP, UK Biobank	19495	258943	278438	71	OR	SD	1.71	1.48	1.97	######	EA***	Yes
BMI, adult	HF	Lind L et al.2021	ULSAM, HERMES, GIANT	405			78	OR		1.68	1.56	1.8	######	European	Yes
BF%	HF	Zhuang et	GIANT, HERMES	47309	930014	977323	10	OR	SD	1.63	1.24	2.13	.16E 0	European	Yes
WC	HF	Lu et al.2023	HERMES, GIANT	47309	930014	977323	12	OR		1.03	0.92	1.14	######	European	NO
BMI, adult	HF	Lu et al.2023	HERMES, GIANT	47309	930014	977323	12	OR		1.74	1.63	1.85	######	European	Yes
WC	HF	Chen et al. 2024	GIANT, HERMES, UK Biobank	47309	930014	977323	249	OR		1.74	1.6	1.9	######	European	Yes
BMI, adult	HF	Chen et al. 2024	GIANT, HERMES, UK Biobank	47309	930014	977323	488	OR		1.7	1.6	1.8	######	European	Yes
FM	HF	Chen et al. 2024	GIANT, HERMES, UK Biobank	47309	930014	977323	295	OR		1.54	1.44	1.65	######	European	Yes
BMI, childhood	HF	Zhou et al.2024	HERMES	47309	930014	977323		OR	SD	1.319	1.16	1.499	######	European	Yes
BMI, adult	HFpEF	Lin et al.2025	Biobank, MVP	23363	187840	211203	30	OR		2.1	1.78	2.47	<0.000	European	Yes
BMI, adult	HFrEF	Lin et al.2025	Biobank, MVP	23363	187840	211203	30	OR		1.85	1.62	2.12	<0.000	European	Yes

BMI, adult	HF	Liu et al.2025	GIANT, Biobank, HERMES			434794	311	OR	SD	1.64	1.54	1.75	< 0.05	European	Yes
WHR	HF	Liu et al.2025	GIANT, Biobank, HERMES			381152	266	OR	SD	1.03	0.97	1.08	< 0.05	European	Yes
BF%	HF	Liu et al.2025	GIANT, Biobank, HERMES			190991	116	OR	SD	1.31	1.17	1.47	< 0.05	European	Yes
VAT	HF	Liu et al.2025	GIANT, Biobank, HERMES			20038	45	OR	SD	0.97	0.86	1.09	< 0.05	European	Yes
ASAT	HF	Liu et al.2025	GIANT, Biobank, HERMES			20038	57.9	OR	SD	0.99	0.88	1.12	< 0.05	European	Yes
FFM	HF	Lu et al.2025	GIANT, Biobank,	23397	194811	218208	424	OR	SD	1.34	1.22	1.47	######	European	Yes
FM	HF	Lu et al.2025	GIANT, Biobank,	23397	194811	218208	340	OR	SD	1.32	1.01	1.73	######	European	Yes
BMI, adult	HF	Lu et al.2025	GIANT, Biobank,	23397	194811	218208	368	OR	SD	1.51	1.18	1.93	######	European	Yes
WC	HF	Lu et al.2025	GIANT, Biobank,	23397	194811	218208	286	OR	SD	1.58	1.12	2.23	######	European	Yes
WHR	HF	Lu et al.2025	GIANT, Biobank, Fi	23397	194811	218208	23	OR	SD	1.25	1.01	1.54	######	European	Yes
BMI, childhood	HF	Wang et al. 2025	Finngen,IEU	29672	357772	387444	16	OR	SD	1.307	1.144	1.494	######	European	Yes
BMI, adult	HF	Zeng et al.2025	GIANT&UK	47309	930014	977323	458	OR	SD	1.736	1.634	1.845	######	European	Yes
WHR	HF	Zeng et al.2025	GIANT&UK	47309	930014	977323	313	OR	SD	1.227	1.127	1.336	######	European	Yes
WHR adj BMI	HF	Zeng et al.2025	GIANT&UK	47309	930014	977323	257	OR	SD	1.006	0.936	1.082	######	European	Yes

Abbreviations: CGPS Copenhagen General Population Study; CCHS Copenhagen City Heart Study; GIANT Genetic Investigation of Anthropometric Traits; HERMES HEart failure Molecular Epidemiology for Therapeutic targetS ; ENGAGE European Network for Genetic and Genomic Epidemiology consortium; ARIC Atherosclerosis Risk in Communities Study; FHS Framingham Heart Study; MESA Multi-Ethnic Study of Atherosclerosis; CHS Cardiovascular Health Study; WHI Women's Health Initiative; EA European American; AA African American; EGG Early Growth Genetics Consortium; CALIBER CArdiovascular disease research using LInked Bespoke studies and Electronic health Records;MVP Million Vet- erans Program ;ULSAM Uppsala Longitudinal Study of Adult Men; CHARGE-HF Cohorts for Heart and Aging Research in Genomic Epi-demiology-Heart Failure Working Group; AAAGC African Ancestry Anthropometry Genetics Consortium; HF Heart Failure; ASAT Abdominal Subcutaneous Adipose Tissue volume;VAT Visceral Adipose Tissue volume;BF% Bdy Fat percentage; FM Fat Mass; BMI Body Mass Index;WHR Waist-to-Hip Ratio; WC Waist Circumference; FFM Fat-fFee Mass;HDL-C High-Density Lipoprotein Cholesterol; LDL-C Low-Density Lipoprotein Cholesterol; T2DM Type 2 Diabetes; TG Triglycerides;HFpEF Heart fFilure with Preserved Ejection Fraction; HFrEF Heart Failure with Reduce Ejection Fraction.

†Childhood body size was categorized into three groups, thinner, about average, and plumper, based upon a question to participants. For comparability purposes, adult body size was represented by a categorical variable comprising 3 levels of being "thinner" (BMI 21.1 -25), "about average" (BMI 25-31.7), and "plumper" (BMI 31.7-59.9). OR represents changes in odds ratio from one level to next.

Leyden investigated collider bias.

***Childhood obesity is defined as a BMI greater than or equeal to the 95th percentile at any point before the age of 18 years. Controls, defined as those with a BMI c*

[illegible] extracted information on self-reported ethnicity and race from questions 4 and 5, respectively, from the MVP baseline survey administered to participants as a part of enrollment. For genetically-inferred ancestry, we ran the program ADMIXTURE 14 in the supervised mode using five sub-populations from the 1000 Genomes Phase 3 dataset 9 . The five reference populations are: CHB for Han Chinese in Beijing, GBR for British in England and Scotland, LWK for Luhya in Webuye, PEL Peruvians from Lima, YRI Yoruba in Ibadan, Nigeria. They represent East Asia, Europe, Eastern Africa, America, and Western Africa, respectively. We compared groups of individuals self-identifying as "White" or "White, non-Hispanic" to the fraction of their ancestry that aligned with the GBR reference populations. On top of the phenotypic definition, we retained samples with > 50% of their genome aligning with GBR for genetically European ancestry and samples with > 50% of their genome aligning with LWK or YRI for genetically African ancestry."

****Both self-reported ethnicity and genetic ancestry were utlized to define NON-Hispanic European American and Non-Hispanic African American particpants in the MVP.*

††Ancestry-specific genetic risk scores of BMI (GRSBMI)

Table 4: Study characteristics of multivariable MR studies included of systematic review

Obesity-related Traits	Disease Outcome	Reference	Data Sources	Cases	Non-cases	Total, N	SNPs	Effect Measure	Unit	Effect Size	LCI	UCI	P	Population	Causal Conclusion
BMI, childhood	Chronic HF	Li et al. 2024	ebi-a-GCST90002409, ebi-a-GCST90018806			525780	16	OR		0.99	0.9	1.1	0.824	European	No
BMI, adult	Chronic HF		ieu-b-40, ebi-a-GCST90018806			1167435	486	OR		1.6	1.44	1.78	<0.001	European	Yes
WC††	HF	Hong et al. 2024	GIANT	47309	930014	1209424	38	OR		1.09	0.88	1.35	0.439	European	No
WHR††	HF		GIANT	47309	930014	1189567	28	OR		1.13	0.96	1.35	0.149	European	No
FM††	HF		UK Biobank	47309	930014	1431460	360	OR		1.16	0.92	1.47	0.203	European	No
FFM††	HF		UK Biobank	47309	930014	1432173	486	OR		0.99	0.87	1.13	0.894	European	No
VAT††	HF		UK Biobank	47309	930014	1010183	5	OR		1.06	0.94	1.2	0.322	European	No
ASAT††	HF		UK Biobank	47309	930014	1010183	2	OR		0.98	0.88	1.1	0.794	European	No
BMI†††	HF	Hong et al. 2024	GIANT	47309	930014	1658598	448	OR		1.59	1.32	1.92	<0.001	European	Yes
WHR†††	HF		GIANT	47309	930014	1189567	28	OR		1.03	0.8	1.33	0.796	European	No
FM†††	HF		UK Biobank	47309	930014	1431460	360	OR		1.39	1.2	1.62	<0.001	European	Yes
FFM†††	HF		UK Biobank	47309	930014	1432173	486	OR		1	0.87	1.15	0.971	European	No
VAT †††	HF		UK Biobank	47309	930014	1010183	5	OR		0.82	0.63	1.06	0.131	European	No
ASAT†††	HF		UK Biobank	47309	930014	1010183	2	OR		1.28	0.93	1.77	0.135	European	No
BMI, childhood	HF	Xiong et al. 2024													
Adjusted for adult BMI	HF		Vogelezang et al, 2020, FinnGen	13087	195091	247798	16	OR	SD	1.41	1.3	1.51	<0.001	European	Yes
Adjusted for LDL-C	HF		Vogelezang et al, 2020, FinnGen	13087	195091	247798	16	OR	SD	1.18	1.03	1.33	<0.05	European	Yes
Adjusted for HDL-C	HF		Vogelezang et al, 2020, FinnGen	13087	195091	247798	16	OR	SD	1.24	1.09	1.39	<0.01	European	Yes
Adjusted for TG	HF		Vogelezang et al, 2020, FinnGen	13087	195091	247798	16	OR	SD	1.2	1.07	1.34	<0.01	European	Yes
Adjusted for Hypertension	HF		Vogelezang et al, 2020, FinnGen	13087	195091	247798	16	OR	SD	1.28	1.11	1.45	<0.01	European	Yes
Adjusted for T2DM	HF		Vogelezang et al, 2020, FinnGen	13087	195091	247798	16	OR	SD	1.25	1.07	1.43	<0.05	European	Yes
Childhood obesity	HF	Xiong et al. 2024													
Adjusted for adult BMI	HF		EGG, FinnGen	13087	195091	222026	5	OR		1.15	1.1	1.19	<0.001	European	Yes
Adjusted for LDL-C	HF		EGG, FinnGen	13087	195091	222026	5	OR		1.07	0.99	1.15		European	No
Adjusted for HDL-C	HF		EGG, FinnGen	13087	195091	222026	5	OR		1.13	1.06	1.2	<0.001	European	Yes
Adjusted for TG	HF		EGG, FinnGen	13087	195091	222026	5	OR		1.03	0.96	1.11		European	No
Adjusted for Hypertension	HF		EGG, FinnGen	13087	195091	222026	5	OR		1.1	1.01	1.2	<0.05	European	Yes
Adjusted for T2DM	HF		EGG, FinnGen	13087	195091	222026	5	OR		1.11	1.04	1.19	<0.01	European	Yes
Childhood body size†	HF	Power et al. 2021	FinnGen Study	9576	159286	168862	263	OR		0.87	0.69	1.09	0.224	European	No
Adult body size†	HF		FinnGen Study	9576	159286	168862	522	OR		2.58	2.13	3.14	<0.001	European	Yes
dipose-tissue-instrumented BM	HF	Leyden et al. 2022	GIANT, UK Biobank	10155	324243	334398	86	OR	SD	1.05	1.03	1.07	#####	European	Yes
Brain-tissue-instrumented-BM	HF		GIANT, UK Biobank	10155	324243	334398	140	OR	SD	1.07	1.05	1.1	#####	European	Yes
BMI, ault	HF	Lumbers et al. 2020	CALIBER	65918	1476313	1542231	1684	OR	SD	1.64	1.58	1.7	#####	European	Yes
BMI, ault	HF	Lind et al. 2021	ULSAM, HERMES, GIANT	405			78	OR		1.57	1.46	1.68	#####	European	Yes
BMI, ault	HF	Lu et al.	HERMES, GIANT	47309	930014	977323	12	OR		1.58	1.49	1.68	#####	European	Yes
WC	HF	Chen et al. 2024	GIANT, HERMES, UK Biobank	47309	930014	977323	249	OR		0.52	0.3	0.9	0.021	European	Yes
BMI, adult	HF		GIANT, HERMES, UK Biobank	47309	930014	977323	488	OR					0.439	European	NO
FM	HF		GIANT, HERMES, UK Biobank	47309	930014	977323	295	OR		2.05	1.27	3.31	0.003	European	Yes
BMI	HFpEF	in et al. 202	Uk Biobank, MVP	23363	187840	211203	30	Beta		0.85			#####	European	Yes
BMI	HFrEF		Uk Biobank, MVP	23363	187840	211203	30	Beta		0.46			3E-10	European	Yes
BMI, adult	HF	iu et al. 202	UK Biobank, HERMES, GIANT			434794	358	OR	SD	1.58	1.47	1.7	#####	European	Yes
WHR	HF		UK Biobank, HERMES, GIANT			381152	343	OR	SD	1	0.95	1.06	#####	European	NO
BF%	HF		UK Biobank, HERMES, GIANT			190991	260	OR	SD	1.25	1.13	1.38	#####	European	Yes
VAT	HF		UK Biobank, HERMES, GIANT			20038	213	OR	SD	0.99	0.91	1.09	#####	European	NO
ASAT	HF		UK Biobank, HERMES, GIANT			20038	10	OR	SD	1.01	0.85	1.21	#####	European	NO
FM	HF	Lu et al. 2025	GIANT, UK Biobank	23397	194811	218208	340	OR	SD	1.66	1.44	1.91	#####	European	Yes
BMI, adult	HF		GIANT, UK Biobank	23397	194811	218208	368	OR	SD	1.55	1.39	1.73	#####	European	Yes

Abbreviations: GIANT Genetic Investigation of ANthropometric Traits; HERMES HEart failure Molecular Epidemiology for Therapeutic targetS; MVP Million Vet- erans Program; ULSAM Uppsala Longitudinal Study of Adult Men; CALIBER CArdiovascular disease research using LInked Bespoke studies and Electronic health Records; EGG Early Growth Genetics Consortium; GLC Global Lipids Consortium; HF Heart Failure; ASAT Abdominal Subcutaneous Adipose Tissue volume; VAT Visceral Adipose Tissue volume;BF% Bdy Fat percentage; FM Fat Mass; BMI Body Mass Index; WHR Waist-to-Hip Ratio; WC Waist Circumference; FFM Fat-fFee Mass; HDL-C High-Density Lipoprotein Cholesterol; LDL-C Low-Density Lipoprotein Cholesterol; 2DM Type 2 Diabetes; TG Triglycerides; HFpEF Heart fFilure with Preserved Ejection Fraction; HFrEF Heart Failure with Reduce Ejection Fraction.

†Childhood body size was categorized into three groups, thinner, about average, and plumper, based upon a question to participants. For comparability purposes, adult body size† was represented by a categorical variable comprising 3 levels of being "thinner" (BMI 21.1 -25), "about average" (BMI 25-31.7), and "plumper" (BMI 31.7-59.9). OR represents changes in odds ratio from one level to next.

†† multivariable MR analysis, adjusted for body mass index

†††multivariable MR analysis, adjusted for waist circumference

Table 5: SD of BMI calcualtion for UK Biobank participants.

Characteristics of UK Biobank participants [27]					Estimated Value	
**Gender**	**Age (45- 54)**	**Mean (SD)**	**Age (55- 64)**	**Mean (SD)**	**Combined Mean**	**SD**
Men	61,860	27.8 (4.4)	94,776	27.9 (4.3)	27.5	4.9
Women	79,714	26.9 (5.4)	116,303	27.3 (5.1)		
